# Hyperspectral differentiation of three grapevine yellows diseases and symptomatically similar stresses

**DOI:** 10.3389/fpls.2026.1794713

**Published:** 2026-03-19

**Authors:** Elias Alisaac, Xiaorong Zheng, Benedikt Fischer, Barbara Jarausch, Pascal Gauweiler, Urban Spitaler, Katrin Janik, Luca Fontanesi, Robin Gruna, Jürgen Beyerer, Michael Maixner, Reinhard Töpfer, Oliver Trapp, Anna Kicherer

**Affiliations:** 1 Julius Kuehn-Institute, Federal Research Centre for Cultivated Plants, Institute for Grapevine Breeding Geilweilerhof, Siebeldingen, Germany; 2 Fraunhofer Institute of Optronics, System Technologies and Image Exploitation IOSB, Karlsruhe, Germany; 3 Julius Kuehn-Institute, Federal Research Centre for Cultivated Plants, Institute for Plant Protection in Fruit Crops and Viticulture, Geilweilerhof, Siebeldingen, Germany; 4 Laimburg Research Centre, Auer (Ora), South Tyrol, Italy

**Keywords:** Bois noir, Flavescence dorée, leafhopper, nutrient deficiencies, palatinate grapevine yellows, virus

## Abstract

**Purpose:**

The study aims to develop a method for detecting and discriminating grapevine yellows (GY) diseases, including Flavescence dorée (FD), Bois noir (BN), and Palatinate grapevine yellows (PGY), using hyperspectral imaging (HSI). Specifically, it seeks to address the challenge of symptom misclassification among visually similar diseases, such as distinguishing between GY diseases and other biotic and abiotic stresses.

**Methods:**

Hyperspectral images of detached leaves from field with GY diseases, Grapevine Leafroll-associated Virus (V), leafhopper (LH) infestation, iron deficiency (Fe), or magnesium deficiency (Mg) were acquired using a mobile platform in the laboratory or in the field. The images were taken in the spectral range of 400-1,000 nm. Classification models were trained on the mean spectra of the leaves to distinguish between different classes.

**Results:**

The model achieved F1-scores ranging from 54.2% to 96.8% for white cultivars and exceeded 95% for all six classes for black cultivars. When limited to GY classes and nonsymptomatic leaves, the F1-scores were 89.8%, 78.7%, 63.4%, and 53.4% for nonsymptomatic, FD, PGY, and BN, respectively.

**Conclusion:**

The study demonstrates the feasibilityof using HSI to detect and discriminate different biotic and abiotic stresses on grapevine leaves, including distinguishing between symptom-similar GY diseases. The developed mobile platform and classification models show promise for largescale monitoring and diagnosis of GY diseases, potentially improving disease management and reducing the risk of symptom misclassification.

## 1 Introduction

Phytoplasmas are phloem-restricted pathogens of economic importance in viticulture, causing grapevine yellows (GY) diseases and severe yield loss (Pagliari and Musetti 2019). There are different phytoplasmas associated with GY diseases in Europe: Flavescence dorée (FD), caused by FD phytoplasma (phylogenetic subgroups 16SrV-C/-D), Bois noir (BN) caused by ‘*Candidatus* Phytoplasma solani’ (CaPsol, phylogenetic subgroup 16SrXII-A), and Palatinate grapevine yellows (PGY) caused by non-FD strains of the phylogenetic subgroup 16SrV-C. These pathogens mainly affect leaves, shoots, flowers, and bunches, often causing symptoms limited to some shoots. Affected shoots have short internodes, remain green due to poor lignification, and develop black pustules. Leaves on affected shoots turn yellow or golden in white cultivars and red to purple in black cultivars, often rolling downward and becoming triangular in some cultivars (Kölber, 2011; Zambon et al., 2018).

Among them, FD phytoplasma is a European quarantine pest capable of epidemic spread in vineyards via the leafhopper *Scaphoideus titanus* (Malembic-Maher et al., 2020). It has been reported in several countries (i.e. Austria, Croatia, France, Hungary, Italy, Portugal, Serbia, Slovenia, Spain, and Switzerland) but is not yet widely established in Germany, where a single case was quickly eradicated (Jeger et al., 2016; Jarausch et al., 2021). The first appearance of *S. titanus* in Germany in 2024 has considerably increased the risk of an FD outbreak (Askani et al., 2024; Jarausch et al., 2025). Recently, in November 2025, vines infected with FD were detected for the first time in the German districts of Lörrach, Breisgau-Hochschwarzwald, and Ortenaukreis (Markheiser, 2025). Therefore, rapid and accurate screening is essential, and there is an urgent need to detect and eliminate infected vines. Phytosanitary regulations also require systematic monitoring of disease incidence in the vineyards, which relies on visual assessment. However, accurate visual identification of FD and other GY diseases is very time-consuming and demands skilled inspectors, as the symptom expression varies among cultivars and can be confused with symptoms caused by other biotic (e.g. virus infection or leafhopper *Empoasca vitis* damage) or abiotic stresses (e.g. Mg- and Fe-deficiency) on grapevines. Moreover, the molecular diagnosis using PCR or qPCR, while more specific, is laborious, time-consuming, and expensive.

Since most plant pathogens interact with their hosts in ways that lead to biochemical and biophysical modifications, leaf spectral patterns change upon infection and during symptom development (Mahlein, 2016). These alterations open up the possibility of applying various sensor technologies to accelerate the detection process. Various imaging and spectral sensing technologies have been explored for the detection of GY and other grapevine diseases under both controlled and field conditions (Singh et al., 2022; Ferro and Catania, 2023). Convolutional neural networks have been successfully trained to classify healthy, GY, and non-GY diseased detached leaves using images (Cruz et al., 2019). RGB cameras have been used for the automatic detection of FD across different grapevine cultivars under field conditions (Tardif et al., 2022, Tardif et al., 2023). However, Boulent et al. (2020) reported that the performance of RGB-based models varied across cultivars due to differences in FD symptom expression. To gain more spectral information, multispectral imaging (MSI) has been used and demonstrated high accuracy in detecting FD under controlled conditions (Hruška et al., 2019; Ryckewaert et al., 2023) and in the field (Barjaktarovic et al., 2023). MSI sensor capturing reflectance at VIS (670 nm), RedEdge (730 nm), and NIR (775 nm) wavelengths enables the calculation of vegetation indices such as the normalized difference vegetation index (NDVI) and normalized difference red edge index (NDRE), which have proven effective for detecting FD or Esca symptoms in vineyards (Daglio et al., 2022). Moreover, MSI sensors mounted on unmanned aerial vehicles (UAV) have been applied to detect FD symptoms in vineyards from above (Albetis et al., 2017; Musci et al., 2020). Hyperspectral imaging (HSI) provides even higher spectral resolution and has shown great potential in distinguishing between healthy, FD-infected, and other diseased grapevine leaves. Al-Saddik et al. (2017; Al-Saddik et al., 2018; Al-Saddik et al., 2019), Sinha et al. (2019) used a non-imaging portable spectroradiometer (350–2,500 nm) to successfully detect FD, Esca, and viral diseases in the field, identifying key wavelength regions relevant for symptom expression. Bendel et al. (2020a) further demonstrated the potential of HSI (400–2,500 nm) for detecting BN and PGY in both greenhouse-grown and field-collected grapevine shoots. In addition, Imran et al. (2023) showed that still asymptomatic FD leaves in an early stage could be distinguished from healthy ones using two low-cost, portable mini-spectrometers operating in the 340–850 nm and 900–1,700 nm ranges, achieving promising classification accuracy under field conditions.

However, no existing model is currently available that effectively addressed the issue of symptom misclassification caused by the visual similarities between various grapevine diseases—such as different types of GYs, viral infections, nutrient deficiencies, and insect-related damage. To address this challenge, the present study aims to:

- Assess the feasibility of combing HSI with machine learning (ML) to differentiate between FD, BN, and PGY symptoms on grapevine leaves under controlled conditions;
- Evaluate the feasibility of HSI and ML to distinguish between different biotic and abiotic stress symptoms (GYs, Grapevine Leafroll-associated Virus, leafhopper infestation, Fe- and Mg-deficiency) on grapevine leaves under controlled conditions;
- Identify the most important wavelengths associated with these stress symptoms to develop a reliable, field-applicable MSI system for disease detection in vineyards.

## 2 Materials and methods

### 2.1 Vineyards and grapevine cultivars

In 2022 and 2023 vineyards affected by different abiotic and biotic stresses in the vicinity of the Institute for Grapevine Breeding, Geilweilerhof, Siebeldingen, Rhineland-Palatinate, Germany, and within plots of the district Unterland in South Tyrol, Italy, were selected for sampling (**Table 1**). In Germany, symptomatic plants in various vineyards were marked in August 2022 or 2023. In September of the respective year, samples (5 leaves per vine per disease) were collected from 20 symptomatic plants and 20 non-symptomatic plants per vineyard. Samples in South Tyrol were taken accordingly in 2023, with the number of leaves varying depending on the onset of symptoms.

**Table 1:** The vineyards sampled for detecting of different biotic and abiotic stresses using hyperspectral imaging technology in Germany and Northern Italy in 2022 and 2023.

Location and year	GPS coordinates	Initials	Plot	Cultivar	Variety number VIVC	Color of berry skin	Symptoms
Albersweiler 2022	49°13’8.2”N 8°01’47.9”E	AW	01	Cabernet Dorsa	20002	Black	BN
Albersweiler 2022	49°13’8.2”N 8°01’47.9”E	AW	02	Dornfelder	3659	Black	BN
Albersweiler 2023	49°13’8.2”N 8°01’47.9”E	AW	05	Dunkelfelder	3724	Black	Mg
Birkweiler 2022	49°12’17.4”N 8°02’13.3”E	BW	02	Pinot Noir	9279	Black	V
Burrweiler 2022	49°14’52.7”N 8°04’33.7”E	BU	02	Riesling Weiss	10077	White	BN
Burrweiler 2022	49°14’52.7”N 8°04’33.7”E	BU	02	Riesling Weiss	10077	White	Mg
Burrweiler 2022	49°14’52.7”N 8°04’33.7”E	BU	04	Pinot Meunier	9278	Black	BN
Frankweiler 2022	49°13’40.1”N 8°03’29.0”E	FW	01	Bacchus Weiss	851	White	Mg
Frankweiler 2022	49°13’40.1”N 8°03’29.0”E	FW	02	Dunkelfelder	3724	Black	LH
Geilweilerhof 2022	49°13’33.6”N 8°02’37.5”E	GF	37	Aligote	312	White	V
Geilweilerhof 2022	49°13’11.8”N 8°02’55.5”E	GF	83	Riesling Weiss	10077	White	LH
Ilbesheim 2023	49°41’27.0”N 8°04’34.6”E	IL	01	Chardonnay Blanc	2455	White	V
Leinsweiler 2022	49°10’57.0”N 8°01’13.8”E	LW	02	Scheurebe	10818	White	BN/PGY
Leinsweiler 2022	49°10’57.0”N 8°01’13.8”E	LW	03	Kerner	6123	White	BN/PGY
Leinsweiler 2022	49°10’57.0”N 8°01’13.8”E	LW	08	Scheurebe	10818	White	BN/PGY
Siebeldingen 2022	49°12’54.7”N 8°03’06.2”E	SB	02	Pinot Noir	9279	Black	Fe
Siebeldingen 2022	49°13’09.7”N 8°03’32.5”E	SB	03	Pinot Blanc	9272	White	Fe
Salurn/Salorno 2023	46°14’20.5”N 11°12’45.5”E	IT	01	Pinot Gris	9275	Gris	FD/BN
Salurn/Salorno 2023	46°14’20.5”N 11°12’45.5”E	IT	02	Pinot Gris	9275	Gris	FD/BN
Salurn/Salorno 2023	46°14’20.5”N 11°12’45.5”E	IT	03	Pinot Gris	9275	Gris	FD/BN
Neumarkt/Egna 2023	46°18’50.7”N 11°16’21.6”E	IT	04	Chardonnay Blanc	2455	White	FD/BN
Salurn/Salorno 2023	46°14’20.5”N 11°12’45.5”E	IT	05	Chardonnay Blanc	2455	White	FD/BN

Bois noir (BN), Fe-deficiency (Fe), Flavescence dorée (FD), Grapevine Leafroll-Associated Virus-1 (GLRaV-1) or Grapevine Leafroll-Associated Virus-3 (GLRaV-3) (V), Grape leafhopper (*Empoasca vitis*) (LH), Mg-deficiency (Mg), Palatinate grapevine yellows (PGY).

### 2.2 Phytoplasma detection using PCR

#### 2.2.1 DNA extraction from leaves

For phytoplasma detection in the German samples, the total nucleic acid of vines with typical symptoms of phytoplasma infection was isolated from fresh or frozen leaf veins and petioles (120–150 mg) with a modified CTAB method (Doyle and Doyle, 1990; Boudon-Padieu et al., 2003). The final pellet was resuspended in 150 µL TE buffer.

For phytoplasma detection in the Italian samples, the DNeasy Plant Mini Kit (Qiagen, Hilden, Germany) was used to extract the DNA, using an adjusted protocol: Small pieces of resh leaf stalks (50–100 mg) were placed in 800 µL CTAB buffer (2.5% w/v Cetyltrimethylammonium-bromide, 100 mM Tris-HCl pH 8.0; 1.4 M NaCl, 50 mM EDTA pH 8.0, 1% w/v Polyvinylpyrrolidon-40, 0.5% w/v freshly added ascorbic acid) containing two tungsten beads 30 mm and homogenized using a TissueLyser II at 30 Hz for 5–10 min. To degrade RNA, 4 µL of RNase A (100 mg/mL) were added to each sample, and the mixture was incubated at 65°C for 30 min. After adding 260 µL of Buffer P3, samples were incubated on ice for 10 min and then centrifuged at 20,000 x g for 10 min. The supernatant was transferred to a QIA shredder column, centrifuged for 2 min at 20,000 x g, and the cleared lysate was collected. The lysate was mixed with 750 µL of Buffer AW1 and transferred to a DNeasy Mini spin column. Columns were centrifuged for 1 min at 6,000 x g, and the flow-through was discarded. The columns were washed by adding 500 µL of Buffer AW2 and centrifugation for 60 s at 6,000 x g. Subsequently, a second wash step was performed by adding 500 µL of 96% v/v ethanol and centrifugation at 20,000 x g for 3 min. To elute the DNA from the column, 100 µL of pre-heated (65°C) AE buffer was added to each column and incubated for 5 min at room temperature. The column was then centrifuged for 1 min at 6,000 x g. This elution step was repeated with a second 100 µL aliquot of AE buffer to recover a total volume of 200 µL of purified DNA per sample.

#### 2.2.2 Detection of phytoplasma using PCR and multiplex qPCR

The simultaneous detection of 16SrV group phytoplasmas including FD and FD-related phytoplasmas, and the 16SrXII group phytoplasmas including the Stolbur phytoplasma (*Ca*. *P. solani*) in the German samples was carried out using the commercial Qualiplante kit in a triplex real-time Taqman^®^ assay (Qualiplante SAS, rue du Mas de Lépôt, Lavérune, France) including an endogenous control (COX gene). The test for the 16SrV group was based on the rpl14 gene sequences and reactions were performed on a 7500 Fast PCR System Applied Biosystems™ (Fisher Scientific GmbH, Schwerte, Germany) in a final volume of 20 µL. Generic phytoplasma detection was carried out by PCR with the universal primers U5/P7 (Lorenz et al., 1995; Schneider et al., 1995), using a 20 µL reaction volume with a final concentration of 0.025 U/µL DreamTaq Green DNA-Polymerase (Thermo Fisher Scientific, Waltham, USA), 0.2 mM dNTPs, 0.5 µM (each primer), 1X DreamTaq Green Buffer (Thermo Fisher Scientific, Waltham, USA), and 2 µL template DNA. Cycle conditions were 2 min at 94°C followed by 5 cycles of (30 s at 94°C, 30 s at 59°C and 90 s at 72°C), 5 cycles of (30 s at 94°C, 30 s at 58°C and 90 s at 72°C), 20 cycles of (30 s at 94°C, 30 s at 57°C and 90 s at 72°C) and a final elongation step of 30 s at 72°C. To identify phytoplasmas of the 16SrV phylogenetic group, PCR was carried out with the group-specific primers fAY/rEY as described by Maixner and Reinert (1999).

A quantitative multiplex PCR approach was performed for the simultaneous detection and quantification of FD- and BN-specific DNA in DNA samples from grapevine leaves in Italy. The used method is a hydrolysis probe-based multiplex qPCR combining primers and probes for FD and BN detection (Hren et al., 2007), and primers and a probe for the detection and quantification of host DNA (Mittelberger et al., 2020). Every sample was analyzed in duplicate. Each reaction was carried out with a 10 µL total volume, using final concentrations of 1X iQ Multiplex Powermix (Bio-Rad, Hercules, CA, USA) and different concentrations of all specific primers (primers for 28S internal control: 0.4 µM/primer and 0.2 µM probe; primers for FD and detection: 0.9 µM/primer and 0.2 µM probe). For the analysis, 8 µL of the master mix containing the above-mentioned components were combined with 2 µL DNA sample. The PCR conditions involved an initial denaturation at 95°C for 3 min, followed by 45 cycles of 95°C denaturation for 15 s, and 60°C annealing and elongation for 1 min. Different fluorescent probes were used, enabling the simultaneous detection and quantification of the target phytoplasmas and host DNA (FAM/BHQ1 for FD, Cy5/BHQ3 for BN and HEX/BHQ1 for host DNA). For an absolute template quantification, plasmids (pJET1.2-FD, pJET1.2-BN, and pJET1.2-28S) were used to generate standard curves with plasmid copy numbers ranging from 10^5^ to 10¹ copies per reaction.

To ensure consistent interpretation of qPCR results and support downstream analyses, leaf samples were classified into four categories [negative, positive (high), positive (low), and positive (very low)] based on amplification behavior and quantification thresholds.

The lowest quantifiable standard was determined using serial plasmid dilutions analyzed in parallel as reference. The “Very Low” category accounts for stochastic effects inherent to PCR at low template concentrations where random distribution of very few target molecules may result in inconsistent amplification and failure to generate curves in one of the replicates (Weusten and Herbergs, 2012).

### 2.3 Virus detection using RT-PCR

#### 2.3.1 RNA extraction from leaves

A mixed sample of 100 mg was taken from five leaves collected from one plant. Leaf material was sampled using a hand cork borer of 1 cm diameter, collected in a 2 mL safe-lock Eppendorf tube that contains 0.04 g of PVP40 powder. The tubes were submerged immediately in liquid nitrogen and stored at -20°C until RNA extraction. The total RNA was extracted from leaf samples using a NucleoSpin^®^ RNA Plant kit (MACHEREY-NAGEL GmbH & Co. KG, Düren, Germany) following the manufacturer’s instructions.

#### 2.3.2 cDNA synthesis and RT-PCR

The cDNA was synthesized using Applied Biosystems™ High-Capacity cDNA Reverse Transcription Kit (Fisher Scientific GmbH, Schwerte, Germany). Each reaction contained 4.2 µL nuclease-free water, 2 µL 10X RT Buffer, 2 µL RT Random Primer, 1 µL MultiScribe Reverse Transcriptase (50 U/µL), 0.8 µL 25X dNTP Mix (100 mM), and 10 µL RNA. The reaction mixture was incubated in the PCR machine as the following: 10 min at 25°C, 120 min at 37°C, 5 min at 85°C and stored at 4°C.

A PCR test was performed on the cDNA to detect Grapevine Leafroll-associated Virus-1 (GLRaV-1), Grapevine Leafroll-associated Virus-3 (GLRaV-3), Grapevine fanleaf virus (GFLV), and Grapevine Pinot Gris Virus (GPGV), using the specific primers, with an additional primer pair targeting grapevine 18S ribosomal RNA as an internal control (**Table 2**). The PCR reaction (10 µL total volume) contained 5 µL of 2× KAPA2G Fast Multiplex PCR Kit (Sigma-Aldrich Chemie GmbH, Taufkirchen, Germany), 1 µL primer mix (18S For/Rev at 0.625 µM each and one virus-specific primer pair For/Rev at 5 µM each), 2.5 µL water, and 1.5 µL cDNA. The PCR was performed according to the instruction of the KAPA2G Fast Multiplex PCR Kit manufacturer starting with an initial denaturation for 3 min at 95°C followed by 30 cycles as the following: 15 s at 95°C, 30 s at 60°C, 30 s at 72°C followed by final extension for 3 min at 72°C.

**Table 2:** The primer pairs used in the RT-PCR to detect virus infections in Germany.

Target	Primer	Sequence 5´-3´	Amplified fragment (bp)	Reference
Plant 18S rRNA (internal control)	18S For 18S Rev	CGCATCATTCAAATTTCTGC TTCAGCCTTGCGACCATACT	844	(Gambino and Gribaudo 2006)
Grapevine Leafroll-associated Virus-1	LR1-H70F1 LR1-H70R1	GTTGGTGAATTCTCCGTTCGT ACTTCGCTTGAACGAGTTATAC	382	(Beuve et al., 2013)
Grapevine Leafroll-associated Virus-3	GLRaV3 For GLRaV3 Rev	TACGTTAAGGACGGGACACAGG TGCGGCATTAATCTTCATTG	336	(Gambino and Gribaudo 2006)
Grapevine Fanleaf Virus	GFLV For GFLV Rev	ATGCTGGATATCGTGACCCTGT GAAGGTATGCCTGCTTCAGTGG	118	(Gambino and Gribaudo 2006)
Grapevine Pinot Gris Virus	CP-F2 CP-R2	ATAGCAGTTGAAGGGACCTC AAGCCGTGATAGCATTAGTC	430	(Bertazzon et al., 2016)

Gel electrophoresis was done on 3% agarose gel (Biozym Scientific GmbH, Hessisch Oldendorf, Germany) in 1× TAE buffer. The gel was stained with Serva DNA Stain Clear G (SERVA Electrophoresis GmbH, Heidelberg, Germany). Ten µL of PCR products were stained with 1.5 µL Gel Loading Dye, Purple (6X) (New England Biolabs GmbH, Frankfurt am Main, Germany) and loaded on the gel. Quantitas Pro DNA Marker 50 bp – 1.5 kb (Biozym Scientific GmbH, Hessisch Oldendorf, Germany) were loaded on the gel to confirm the size of the amplified fragment of the virus. After gel electrophoresis, the gel was imaged under UV light.

### 2.4 Quantification of Mg and Fe content in grapevine leaves using AAS

Five leaves were collected from a single plant, placed in a paper bag and lyophilized using Alpha 1–4 LSC Laboratory freeze-dryer (Martin Christ Gefriertrocknungsanlagen GmbH, Osterode am Harz, Germany) at -25°C and 0.310 mbar for 48 h.

To digest the sample for Mg quantification, 300 mg grounded dry leaf powder were mixed with 8 mL of Nitric acid HNO_3_ (65%) (CHEMSOLUTE^®^ Laboratory chemicals, Renningen, Germany) and heated in Mars One microwave (CEM GmbH, Kamp-Lintfort, Germany) at 800 watts, maintained at 190°C for 20 min, and cooled to 70°C. Samples were transferred to volumetric flasks, and diluted to 50 mL with distilled water. Blank sample was prepared in parallel for each run. When the dilution was necessary, Mg filtrate was diluted with Cesium Chloride CsCl (1 g/L) (ApplieChem GmbH, Darmstadt, Germany). The calibration series was prepared with acidified CsCl solution (20 mL of nitric acid 65% for 1 L CsCl (1 g/L)). Multielement standard solution 3, with Mg concentration of 400 mg/L, for ICP (Sigma-Aldrich Chemie GmbH, Buchs, Switzerland) was used to prepare 1:100, 1:143, 1:250, and 1:500 calibration series with 4.0, 2.8, 1.6, and 0.8 mg/L, respectively.

To prepared the samples for Fe quantification, 500 mg of dry leaf powder and blank sample were treated as described above. When a dilution was necessary, Fe filtrate was diluted with distilled water. The calibration series was prepared with acidified water (20 mL of nitric acid 65% for 1 L water). Iron standard for ICP (Fe, 1000 mg/L; Sigma-Aldrich Chemie GmbH, Buchs, Switzerland) was used to prepare 1:250, 1:350, 1:500, 1:750, and 1:1000 calibration series with 4.0, 2.857, 2.0, 1.333 and 1.0 mg/L, respectively.

Flame atomic absorption instrument Agilent 240FS AA (Agilent, Waldbronn Germany) was used for Mg and Fe quantification.

### 2.5 Hyperspectral image acquisition

**Figure 1:**
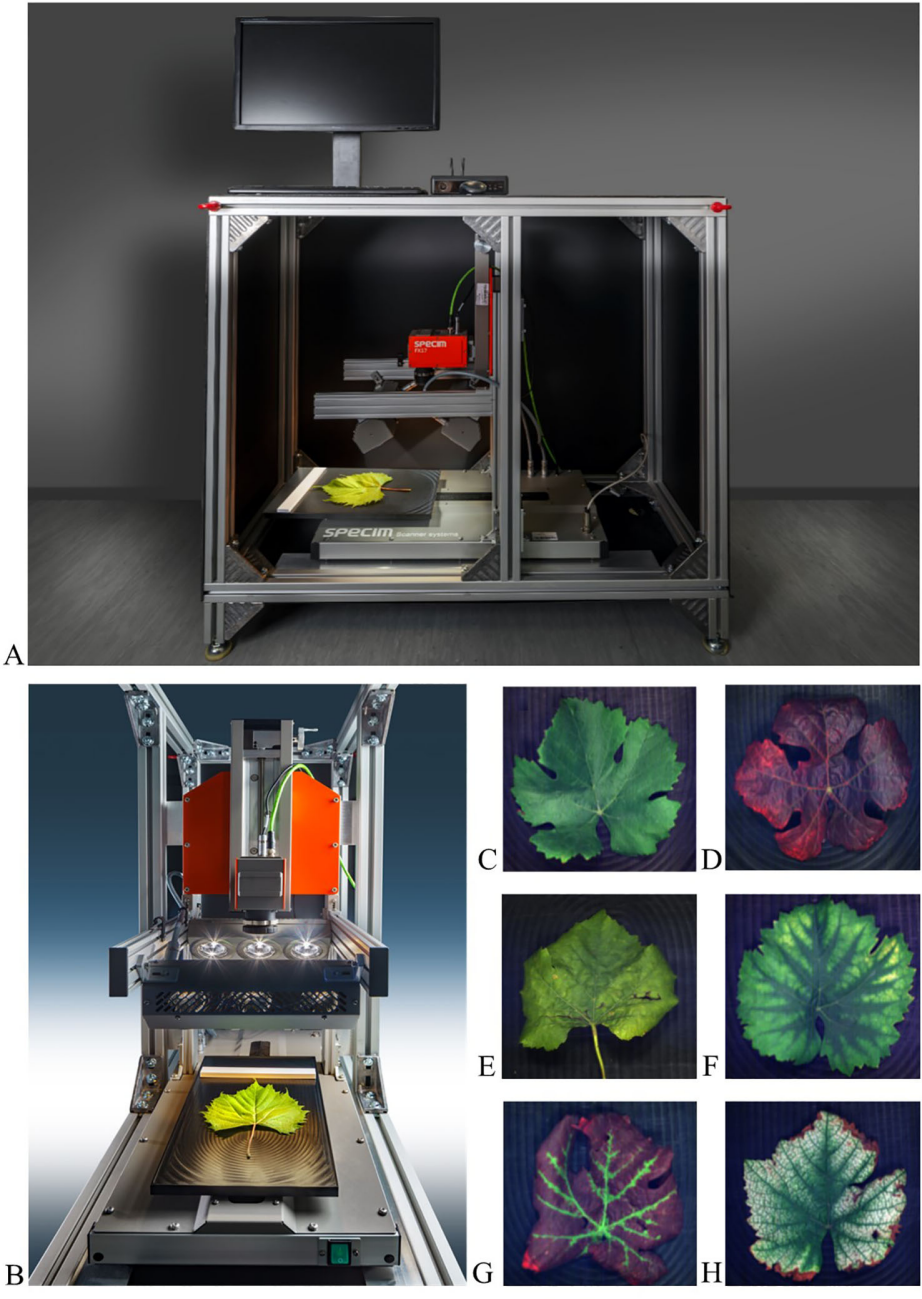
Mobile platform for lab and field image acquisition. **(A)** image acquisition system with HSI camera, illumination, and moving table. **(B)** RGB images of exemplary leaves: **(C)** Non-symptomatic-Pinot Noir, **(D)** Bois noir on Pinot Meunier, **(E)** Flavescence dorée on Pinot Gris, **(F)** Mg-deficiency on Bacchus Weiss, **(G)** Grapevine Leafroll-associated Virus-1 on Pinot Noir, **(H)** Fe-deficiency on Pinot Blanc.


A mobile platform comprising a sensor, a motorized table, and an operation unit was constructed for hyperspectral image acquisition in the laboratory and in the field. The Specim FX10 camera (Spectral Imaging Ltd., Oulu, Finland) with 224 spectral bands in the VIS-NIR wavelength range 400-1,000 nm and a mean spectral resolution of 5.5 nm was mounted on a Specim LabScanner system that includes a linear moving table platform to place the samples on (**Figure 1**). The image acquisition settings were adjusted using the Lumo Scanner software (Spectral Imaging Ltd., Oulu, Finland) as the following: frame rate 60.00 Hz, exposure time 10.00 ms, spectral binning 1, spatial binning 1, and scanning speed 12.73 mm/s. Halogen illumination (Decostar 51 ALU 20W 12V 36° GU5.3) was used to ensure lighting across the whole wavelength range. To get reproducible light conditions, the illumination source and the camera were preheated for 30 min before image acquisition. To correct for system-induced spectral variability, the raw hyperspectral images of the grapevine leaves were normalized using both black and white reference images. The black reference image accounts for the camera’s dark current and system noise and was acquired by closing the shutter of the camera. For the white reference image, a gray reference target that reflects around 54% of the radiated light (SphereOptics, Herrsching, Germany) was recorded. The normalized (white-referenced) hyperspectral image for each pixel was calculated as:

$$I=\frac{I_{raw}-I_{black}}{I_{white}-I_{black}}\times R$$

Where *I_raw_* is the raw pixel intensity for each spectral band, *I_black_* ​ is the intensity of the black reference image, *I_white_* is the intensity of the white reference image, and *R* is the absolute reflectance of the used white reference panel for each spectral band.

In total, 4,500 hyperspectral images of fresh leaf samples were acquired. To ensure a balanced representation of each class, subsampling was performed as detailed in **Supplementary Tables 1**–**S5**.

### 2.6 Image analysis and machine learning approach

**Figure 2:**
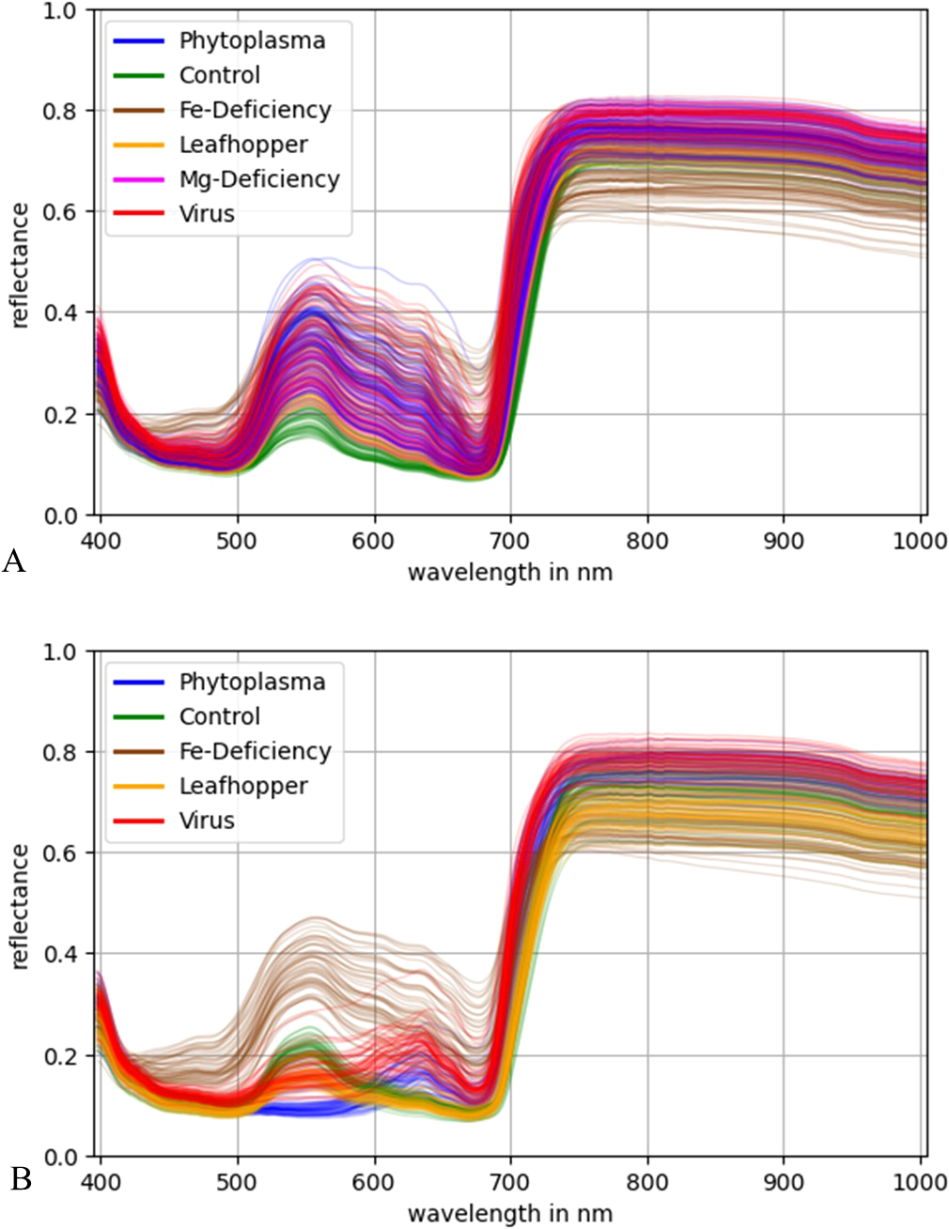
Mean spectral reflectance of the white **(A)** and black **(B)** cultivar leaves colored by their disease.

In the first step, the leaves were segmented from the black background, using a triangle thresholding method on the images where the spectral dimension was reduced to one channel by taking the mean over all channels. From these segmented images, the mean spectral reflectance of each leaf was calculated (**Figure 2**).

For all experiments, a Partial Least Squares Discriminant Analysis (PLS-DA) model was trained. The number of latent variables in the PLS-DA was treated as a hyperparameter and optimized on the validation set; for each experiment, the optimal value was selected from the range [1, 20]. The mean spectra were further preprocessed by different spectral preprocessing methods. A small preliminary study was conducted and showed that Standard Normal Variate (SNV) preprocessing followed by Savitzky-Golay filtering with the first derivative, a polygon order of 3, and a window length of 5 gave the best results. Hence, these preprocessing steps were used in all subsequent experiments.

Depending on the experiment, the number of images used for training and testing was subsampled to get a better-balanced dataset (**Table 3**). The subsampling was performed in a stratified way with respect to both the disease classes and the cultivars. Due to the similarity to the white cultivar, leaves from Pinot Gris were classified as white in both model training and testing.

**Table 3:** Number of images used in model training and testing.

Model	C	BN	PGY	FD	Fe	Mg	LH	V
KP	40	40	20	–	–	–	–	–
SP	60	60	38	–	–	–	–	–
CP	40	18	–	40	–	–	–	–
PP	40	40	–	33	–	–	–	–
WP	80	80	58	80	–	–	–	–
WD	80	80	58	80	80	80	80	80
BD	100	100	–	–	99	99	100	99
ALL	80	80	58	80	80	80	80	80

Non-symptomatic (C); Bois noir (BN); Palatinate grapevine yellows (PGY); Flavescence dorée (FD); Fe-deficiency (Fe); Mg-deficiency (Mg); Grape leafhopper (*Empoasca vitis*) (LH); Grapevine Leafroll-associated Virus (V); Phytoplasma model for Kerner (KP); Phytoplasma model for Scheurebe (SP); Phytoplasma model for Chardonnay Blanc (CP); Phytoplasma model for Pinot Gris (PP); Phytoplasma model for white grapevine cultivars (WP); Disease model for white grapevine cultivars (WD); Disease model for black grapevine cultivars (BD); Disease model for white and black grapevine cultivars (ALL).

For the four experiments on single cultivars (KP, SP, CP and PP in **Table 3**), the absolute number of images per class ranged from 18 to 60. With such small datasets, the choice of the training and test sets has a high influence on the results. Therefore, the data was split in the following way. First, the data was split randomly into 10% test and 90% training. Then a 10-fold cross validation was performed on the 90% training data to get the optimal hyperparameters. The optimal model was then evaluated on the 10% test data. This process was repeated 40 times to get more meaningful test results. From all test results the mean and standard deviation was calculated for the final result.

For the experiments with multiple cultivars (**Table 3**), the data was first split into 25% test and 75% training data. Then a 5-fold cross validation was performed on the 75% training data to get the optimal hyperparameters. The optimal model was then evaluated on the 25% test data. This process was repeated three times and then the mean and standard deviation were calculated. The model hyperparameters are the number of components.

In the phytoplasma specific model (**Table 3**) cultivars and diseases were coupled. For example, PGY disease occurred frequently in Scheurebe and were not present in Chardonnay Blanc and Pinot Gris (**Supplementary Table 1**). To investigate this influence of grapevine cultivars on the disease classification an additional model (WPm) was conducted. The model was first trained only on non-symptomatic control leaves of the four cultivars Kerner, Scheurebe, Chardonnay Blanc and Pinot Gris to distinguish between different cultivars. The importance of individual wavelengths for the cultivar classification was assessed systematically with a permutation feature importance approach. The model was trained on the original data and then its performance was evaluated on the altered test data, where the values of each wavelength and its first neighbors were replaced by linearly interpolating the values from the second-order neighbors. The wavelengths that resulted in a drop in the overall F1-score of more than 10% when replaced, with respect to the F1-score on the unmodified test data, were considered be highly associated with cultivar differentiation. These important cultivar wavelengths were masked and excluded from the train and test data of the disease classification model WPm.

## 3 Results

### 3.1 Phytoplasma incidence in grapevine cultivars

The PCR investigations on the German samples showed that all randomly sampled non-symptomatic plants in the test plots were free from phytoplasmas of the 16SrXII group (BN) or the 16SrV group (FD or PGY) in all tested cultivars. The majority of symptomatic plants from all cultivars was infected by the BN phytoplasma ‘*Candidatus* Phytoplasma solani’ with infection rates up to 95%. The FD-related PGY phytoplasmas were only found in the white cultivars Scheurebe and Kerner (**Table 4**).

**Table 4:** PCR results of phytoplasma detection in six grapevine cultivars in the German test plots in 2022 using specific primers.

Grapevine cultivar	Color of berry skin	Bois noir	Palatinate grapevine yellows
Kerner	White	11	4
Riesling Weiss	White	19	0
Scheurebe	White	12	8
Cabernet Dorsa	Black	19	0
Dornfelder	Black	16	0
Pinot Meunier	Black	16	0

20 symptomatic and 20 non-symptomatic samples were tested. The numbers in the table are the positive reactions out of 20 symptomatic samples.

The qPCR investigations on the Italian samples showed that out of 38 Chardonnay Blanc samples 12 were healthy, 2 had BN infections, and 24 had FD infections ranging between very low and high without any mixed infections. In the case of Pinot Gris, out of 37 samples, 9 samples were healthy, 11 showed BN infections, and 11 had FD infections. In addition, 6 samples showed BN and FD infections simultaneously (**Table 5**). Leaves with a very low phytoplasma level were excluded from model training.

**Table 5:** The qPCR results of phytoplasma detection in two grapevine cultivars in the Italian test plots in 2023 using specific primers and probes.

Grapevine cultivar	Healthy	Bois noir			Flavescence dorée		
		Very low	Low	High	Very low	Low	High
Chardonnay Blanc	12	0	0	2	5	2	17
Pinot Gris	9	0	0	17	8	3	6

38 Chardonnay Blanc samples and 37 Pinot Gris samples were tested. Healthy: no amplification within 45 cycles; High: both replicates above lowest quantifiable standard (FD >200 copies/reaction; BN >40 copies/reaction); Low: both replicates positive but below lowest quantifiable standard (FD <200 copies/reaction; BN <40 copies/reaction); Very low: one replicate positive but below lowest quantifiable standard (FD <200 copies/reaction; BN <40 copies/reaction) and one curve negative.

### 3.2 Virus incidence in grapevine cultivars in Germany

The RT-PCR results showed that all non-symptomatic samples were virus-free in all tested cultivars in 2022 and 2023. The GLRaV-1 and GLRaV-3 infections were confirmed in all tested Pinot Noir and Aligote samples for both years. In the case of Chardonnay Blanc, 3 samples showed GLRaV-1 infection and 8 out of 20 samples tested showed GLRaV-3 infection in the year 2023, without any case of mixed infection (**Table 6**).

**Table 6:** RT-PCR results of virus detection in three grapevine cultivars in the German test plots in 2022 and 2023 using specific primers.

Grapevine cultivar	2022				2023			
	GLRaV-1	GLRaV-3	GFLV	GPGV	GLRaV-1	GLRaV-3	GFLV	GPGV
Aligote	0	20	0	0	0	20	0	0
Chardonnay Blanc	n.t	n.t.	n.t.	n.t.	3	8	0	0
Pinot Noir	20	0	0	0	20	0	0	0

20 symptomatic and 20 non-symptomatic samples were tested. The numbers in the table are the positive reactions out of 20 symptomatic samples. n.t. “not tested”.

### 3.3 Mg and Fe content in grapevine leaves

**Figure 3:**
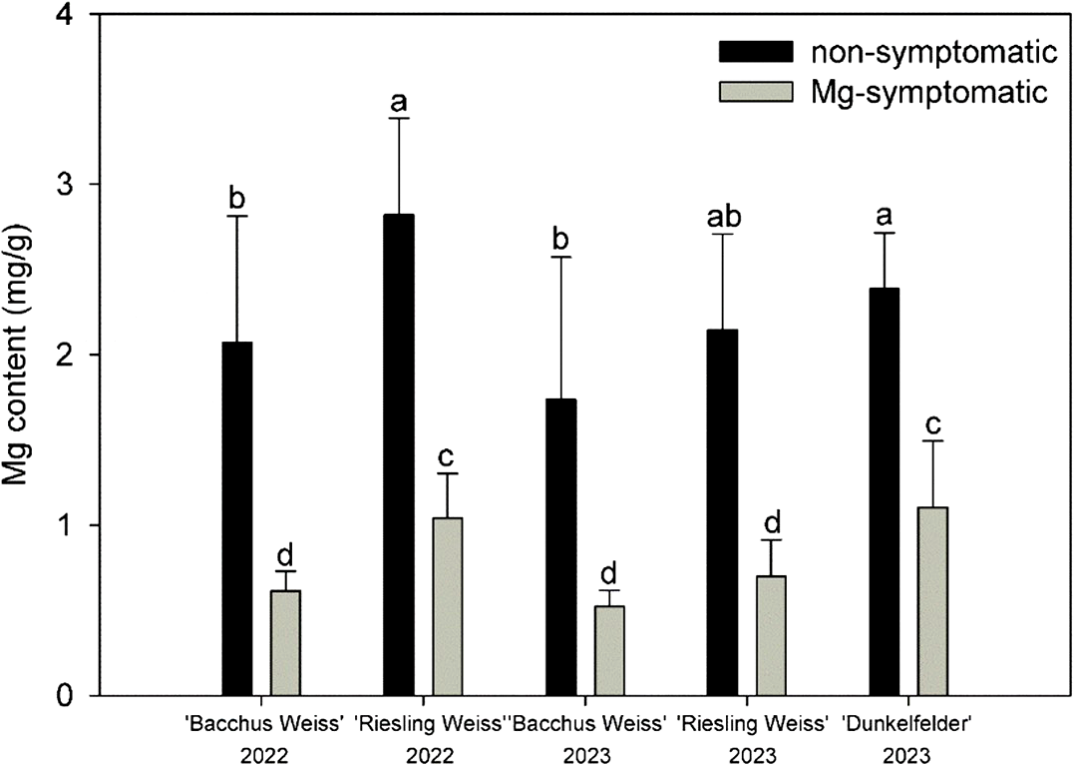
Magnesium content ± SD in symptomatic and non-symptomatic leaf samples of three grapevine cultivars (Bacchus Weiss, Riesling Weiss, and Dunkelfelder) in 2022 and 2023. Kruskal–Wallis test (*p* ≤ 0.0001); n = 20; treatments with the same letters at the level of the year are not significantly different.

In 2022, the Mg content differed significantly between non-symptomatic and symptomatic leaves (**Figure 3**). It ranged between 2.1 ± 0.8 and 2.8 ± 0.6 mg/g in non-symptomatic leaves and 0.6 ± 0.1 and 1.1 ± 0.3 mg/g in symptomatic leaves from Bacchus Weiss and Riesling Weiss, respectively. The same tendency was shown in 2023, and the Mg content was 1.7 ± 0.8, 2.2 ± 0.6, and 2.4 ± 0.3 mg/g in non-symptomatic leaves and 0.5 ± 0.1, 0.7 ± 0.2, and 1.1 ± 0.4 mg/g in symptomatic leaves from Bacchus Weiss, Riesling Weiss, and Dunkelfelder, respectively.

**Figure 4:**
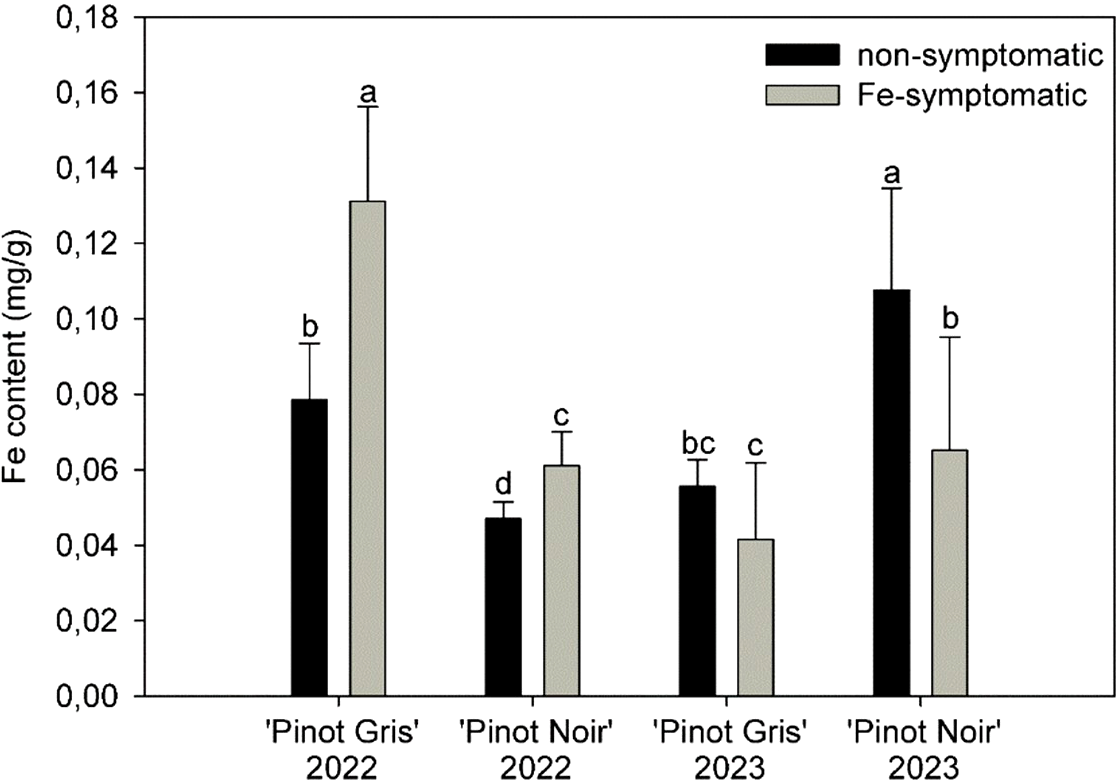
Fe content ± SD in symptomatic and non-symptomatic leaf samples of two grapevine cultivars (Pinot Gris and Pinot Noir) in 2022 and 2023. Kruskal–Wallis test (*p* ≤ 0.0001); n = 20; treatments with the same letters at the level of the year are not significantly different.

In the case of Fe content, non-symptomatic leaves had a significantly lower Fe content compared with symptomatic ones in 2022 with an amount of 0.08 ± 0.1 and 0.05 ± 0.01 mg/g for non-symptomatic leaves and 0.13 ± 0.03 and 0.06 ± 0.01 mg/g for symptomatic leaves from Pinot Blanc and Pinot Noir, respectively (**Figure 4**). However, in contrast to 2022, no significant differences were observed between non-symptomatic and symptomatic leaves in Pinot Gris in 2023, and non-symptomatic leaves in Pinot Noir even showed higher content compared to samples from 2022.

### 3.4 Models for discrimination among different cultivars and stresses

#### 3.4.1 Discrimination among phytoplasmas on different cultivars

The trained models successfully discriminated between the healthy control (C), BN and PGY/FD samples within each individual cultivar. Most of the non-symptomatic leaves (F1-score > 80%) were correctly predicted (**Table 7**). Compared to the differentiation between C, BN, and FD, differentiation between C, BN, and PGY has a higher accuracy with an overall F1-score of 92.5 ± 9.7% in Kerner and 83.3 ± 9.2% in Scheurebe. For the grapevine cultivar Kerner, which achieved the highest F1-score, all 160 images of control leaves were correctly classified, 10 of 160 BN-infected leaves were misclassified as PGY, and 68 of 80 PGY-infected leaves were correctly identified (**Table 8**).

**Table 7:** Harmonic mean of precision and recall (F1-Score in %) for the disease classification models.

Model	C	BN	PGY	FD	Fe	Mg	LH	V	Mean
KP	97.2 ± 4.8	95.9 ± 6.3	84.4 ± 21.4	–	–	–	–	–	92.5 ± 9.7
SP	96.5 ± 5.4	82.4 ± 11.1	71.0 ± 20.0	–	–	–	–	–	83.3 ± 9.2
CP	80.9 ± 11.1	34.5 ± 24.1	–	72.2 ± 17.4	–	–	–	–	62.6 ± 16.9
PP	80.0 ± 16.4	78.3 ± 16.9	–	75.4 ± 19.5	–	–	–	–	77.9 ± 14.0
WP	89.8 ± 7.3	53.4 ± 7.5	63.4 ± 4.0	78.7 ± 4.1	–	–	–	–	71.3 ± 1.6
WPm	83.1 ± 6.8	54.5 ± 3.2	71.4 ± 7.0	62.6 ± 7.7	–	–	–	–	67.9 ± 3.2
WD	87.7 ± 2.5	55.6 ± 5.6	54.2 ± 6.9	76.4 ± 1.7	96.5 ± 2.5	92.4 ± 1.7	95 ± 2.6	96.8 ± 1.2	81.8 ± 2.0
BD	92.4 ± 3.5	96.6 ± 1.0	–	–	100 ± 0.0	100 ± 0.0	93.6 ± 2.3	97.4 ± 1.9	96.7 ± 0.5
ALL	81.7 ± 2.4	57.3 ± 15.0	70.1 ± 10.6	85.9 ± 5.3	95.7 ± 2.4	85.9 ± 3.3	90.3 ± 1.7	92.4 ± 5.7	82.4 ± 4.3

Non-symptomatic (C); Bois noir (BN); Palatinate grapevine yellows (PGY); Flavescence dorée (FD); Fe-deficiency (Fe); Mg-deficiency (Mg); Grape leafhopper (*Empoasca vitis*) (LH); Grapevine Leafroll-associated Virus (V); Phytoplasma model for Kerner (KP); Phytoplasma model for Scheurebe (SP); Phytoplasma model for Chardonnay Blank (CP); Phytoplasma model for Pinot Gris (PP); Phytoplasma model for white grapevine cultivars with whole wavelength range (WP) and without cultivar-relevant wavelengths (WPm); Disease model for white grapevine cultivars (WD); Disease model for black grapevine cultivars (BD); Disease model for white and black grapevine cultivars (ALL). The F1-Scores are shown as mean and standard deviation of the test-set results from the n repetitions of the modeling and evaluation processes (Models KP, SP, CP and PP were repeated 40 times, while other models were repeated 3 times).

**Table 8:** Confusion matrix for the model classification among phytoplasmas on a) Kerner, b) Scheurebe, c) Chardonnay Blanc and d) Pinot Gris.

Kerner					Scheurebe				
**Ground Truth**	C	**160**	0	0	**Ground Truth**	C	**239**	1	0
	BN	0	**150**	10		BN	10	**201**	29
	PGY	10	2	**68**		PGY	8	44	**108**
		C	BN	PGY			C	BN	PGY
**a**		**Prediction**			**b**		**Prediction**		
Chardonnay Blanc					Pinot Gris				
**Ground Truth**	C	**145**	4	11	**Ground Truth**	C	**134**	12	14
	BN	26	**25**	29		BN	20	**125**	15
	FD	30	15	**115**		FD	23	20	**117**
		C	BN	FD			C	BN	FD
**c**		**Prediction**			**d**		**Prediction**		

Samples of Kerner and Scheurebe were collected from Germany, while samples of Chardonnay Blanc and Pinot Gris were collected from Italy. The values shown are the sum of the test-set results from the 40 repetitions of the modeling and evaluation process. Non-symptomatic (C); Bois noir (BN); Palatinate grapevine yellows (PGY); Flavescence dorée (FD).

The bold values indicate the number of images that were correctly predicted.

#### 3.4.2 Discrimination among phytoplasmas in white grapevine cultivars

The WP model achieved the highest F1-score for C at 89.8 ± 7.3%, followed by FD at 78.7 ± 4.1%, while performance was lower for PGY and BN, with F1-score of 63.4 ± 4.0% and 53.4 ± 7.5%, respectively (**Table 7**). This is reflected in the confusion matrix, where 56 of 60 C and 52 of 60 FD were correctly classified, whereas the model struggled to distinguish between BN and PGY (**Table 9**).

**Table 9:** Confusion matrix for the classification models for phytoplasmas in white grapevine cultivars, with whole wavelength range (WP) and without cultivar-relevant wavelengths (WPm).

**Ground Truth**	C	**56**	0	0	4		**Ground Truth**	C	**55**	3	0	2
	BN	1	**28**	15	16			BN	5	**34**	7	14
	PGY	6	11	**28**	0			PGY	2	14	**29**	0
	FD	2	6	0	**52**			FD	11	14	0	**35**
		C	BN	PGY	FD				C	BN	PGY	FD
WP		**Prediction**					WPm		**Prediction**			

Non-symptomatic (C); Bois noir (BN); Palatinate grapevine yellows (PGY); Flavescence dorée (FD). The values shown are the sum of the test-set results from the 3 repetitions of the modeling and evaluation process.

The bold values indicate the number of images that were correctly predicted.

To minimize the influence of cultivar-specific features, wavelengths important for cultivar classification were identified (**Supplementary Figure 1**) and excluded from the WPm model. However, the exclusion slightly reduced F1-score of the cultivar classification from 100% to 98% (data not shown), while the overall F1-score of the WPm model was reduced to 67.9 ± 3.2%, over 3% lower than that of the WP model (**Table 7**). An increased confusion between BN and FD classifications were observed (**Table 9**). The larger relative decline in the WPm model’s performance suggests that the excluded wavelengths were more critical for phytoplasma classification than for distinguishing cultivars.

#### 3.4.3 Discrimination among biotic and abiotic stresses in white grapevine cultivars

The model demonstrated high performance in classifying non-symptomatic leaves, Fe- and Mg-deficiencies, leafhopper, and virus symptoms, as indicated by high F1-scores, while classification of phytoplasma infected leaves (BN, PGY, and FD) was significantly less accurate (**Table 7**). The confusion matrix reveals that most of the false negative BN-leaves were misclassified as PGY or FD, FD false negatives were mainly classified as C and BN, and false negative PGY-leaves were distributed across multiple classes (**Table 10**).

**Table 10:** Confusion matrix for the model classification of various diseases in white grapevine cultivars.

**Ground Truth**	C	**63**	0	0	1	1	0	4	1
	BN	0	**25**	8	26	1	1	1	1
	PGY	7	0	**17**	0	2	7	2	2
	FD	3	1	0	**51**	0	0	0	0
	Fe	0	0	0	0	**56**	0	0	0
	Mg	0	1	0	0	0	**54**	0	0
	LH	0	0	0	0	0	0	**66**	0
	V	0	0	0	0	0	0	0	**63**
		C	BN	PGY	FD	Fe	Mg	LH	V
		**Prediction**							

Non-symptomatic (C); Bois noir (BN); Palatinate grapevine yellows (PGY); Flavescence dorée (FD); Fe-deficiency (Fe); Mg-deficiency (Mg); Grape leafhopper (*Empoasca vitis*) (LH); Grapevine Leafroll-associated Virus (V). The values shown are the sum of the test-set results from the 3 repetitions of the modeling and evaluation process.

The bold values indicate the number of images that were correctly predicted.

#### 3.4.4 Discrimination among biotic and abiotic stresses in black grapevine cultivars

For black grapevine cultivars, a model to discriminate among biotic and abiotic stresses was developed. The model achieved better performance than the one applied to white cultivars, with F1-score exceeding 92% across all classes and a mean F1-score of 96.7 ± 0.5% (**Table 7**). The confusion matrix showed only a few false negatives, most notably 8 control leaves were falsely classified as LH (**Table 11**).

**Table 11:** Confusion matrix for model classification of various diseases in black grapevine cultivars.

**Ground Truth**	C	**67**	0	0	0	8	0
	BN	1	**71**	0	0	0	3
	Fe	0	0	**75**	0	0	0
	Mg	0	0	0	**75**	0	0
	LH	2	0	0	0	**73**	0
	V	0	1	0	0	0	**74**
		C	BN	Fe	Mg	LH	V
		**Prediction**					

Non-symptomatic (C); Bois noir (BN); Fe-deficiency (Fe); Mg-deficiency (Mg); Grape leafhopper (*Empoasca vitis*) (LH); Grapevine Leafroll-associated Virus (V). The values shown are the sum of the test-set results from the 3 repetitions of the modeling and evaluation process.

The bold values indicate the number of images that were correctly predicted.

#### 3.4.5 Discrimination among biotic and abiotic stresses

**Figure 5:**
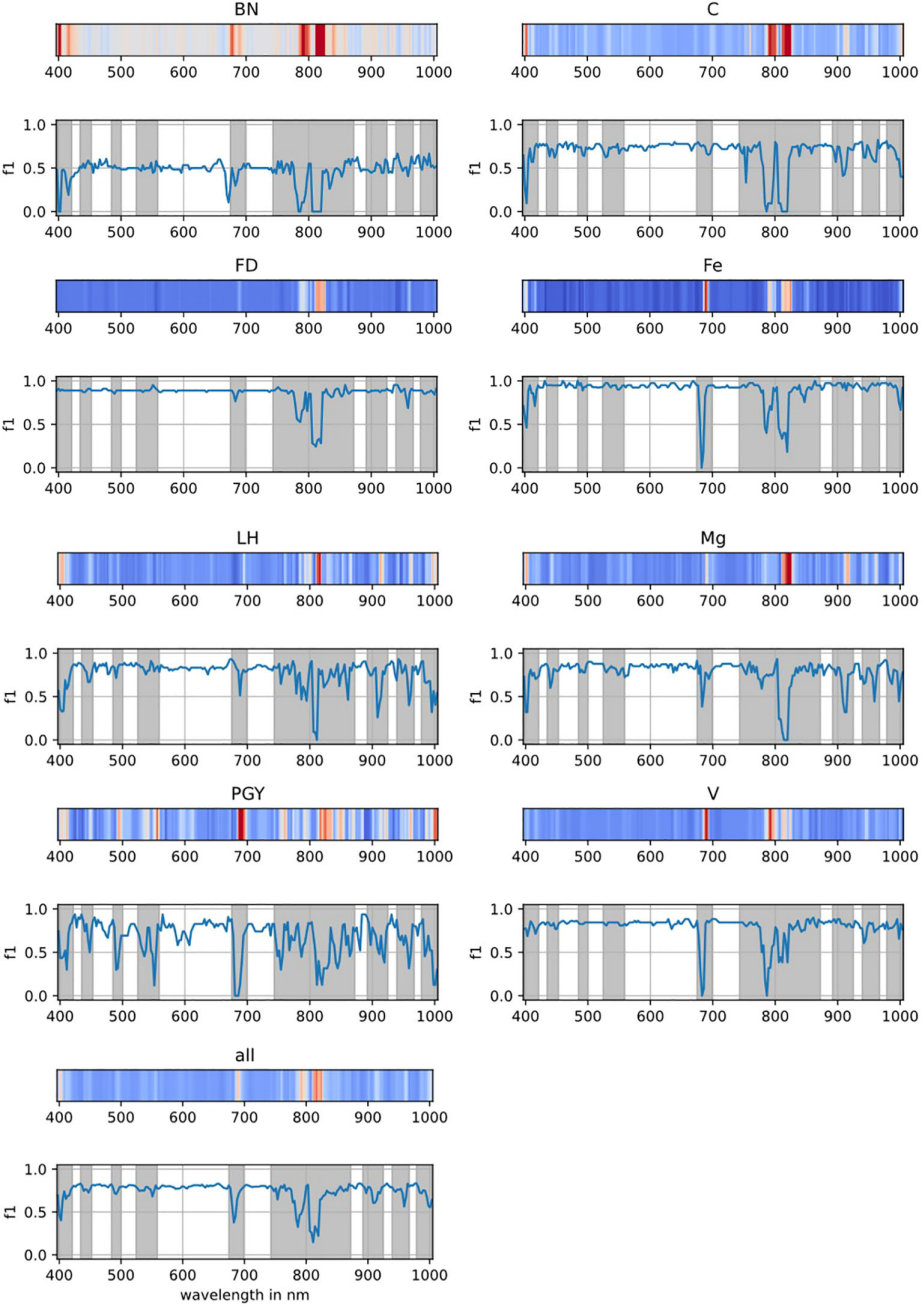
Contribution map of the individual wavelengths for the disease classification performance of the model. The blue line shows the F1-score on the test set when the individual wavelength value was replaced by interpolation from the neighboring wavelengths. The gray area marks the wavelengths where the overall F1-score was reduced by more than 10% with respect to the unmodified data. Non-symptomatic (C); Bois noir (BN); Palatinate grapevine yellows (PGY); Flavescence dorée (FD); Fe-deficiency (Fe); Mg-deficiency (Mg); Grape leafhopper (*Empoasca vitis*) (LH); Grapevine Leafroll-associated Virus (V).

The mean F1-score of this model was 82.4% (**Table 7**). The confusion matrix showed no false negatives for Fe-deficient leaves, while FD and LH each had one false negative misclassified as BN and Fe, respectively (**Table 12**). False negatives for C, BN, PGY, Mg, and V were distributed across other classes. Stress-specific wavelengths contributing most to disease identification included 400–403 nm, 680–686 nm, 784–795 nm, 806–820 nm, 911 nm, 959 nm, and 998–1001 nm (**Figure 5**).

**Table 12:** Confusion matrix for the model classification of various stresses in white and black grapevine cultivars.

**Ground Truth**	C	**57**	0	0	4	0	2	6	1
	BN	5	**27**	11	12	1	3	1	3
	PGY	4	1	**26**	0	3	1	2	0
	FD	0	1	0	**54**	0	0	0	0
	Fe	0	0	0	0	**56**	0	0	0
	Mg	3	0	0	0	0	**46**	3	3
	LH	0	0	0	0	1	0	**65**	0
	V	0	3	0	0	0	0	1	**59**
		C	BN	PGY	FD	Fe	Mg	LH	V
		**Prediction**							

Non-symptomatic (C); Bois noir (BN); Palatinate grapevine yellows (PGY); Flavescence dorée (FD); Fe-deficiency (Fe); Mg-deficiency (Mg); Grape leafhopper (*Empoasca vitis*) (LH); Grapevine Leafroll-associated Virus (V). The values shown are the sum of the test-set results from the 3 repetitions of the modeling and evaluation process.

The bold values indicate the number of images that were correctly predicted.

## 4 Discussion

Since Flavescence dorée (FD) is a quarantine pest in the EU, official control measures are mandatory in all affected member states to maintain low prevalence levels of both the disease and its insect vector (Jeger et al., 2016). However, the recent ban of traditional insecticides has been identified as a key factor contributing to recent outbreaks of the vector and, consequently, the associated phytoplasma disease (Prazaru et al., 2023). The example of South Tyrol illustrates the importance of early detection and timely control, as the continued spread of *S. titanus* since the first detection in 2010 contrast with initially low incidence of FD, likely due to effective measures, such as monitoring, removal of infested vines, and targeted insecticide treatments (Pozzebon et al., 2015; Gallmetzer et al., 2020). Until 2024, neither FD nor its main vector was present in Germany (EPPO, 2024). But during a national monitoring program near selected border crossings with Switzerland and France the first detection of *S. titanus* was reported in Southern Baden in 2024 (Askani et al., 2024). The presence of *S. titanus* leads to an increased risk for an FD outbreak and requires more targeted and efficient controls (Jarausch et al., 2025). In the present study, by carefully managing the training and testing sets in a small-sample experiment, the potential bias from dataset selection was minimized. Consequently, several models for different scenarios of application were successfully developed using HSI sensor system to differentiate symptoms of three types of phytoplasma as well as other biotic and abiotic stresses, and to identify key wavelengths enabling more objective, boarder, and more effective monitoring across entire grapevine growing regions.

Similar to previous studies, the models developed in the present study were able to differentiate among healthy, BN, and PGY in Kerner or Chardonnay, as well as among healthy, BN, and FD in Scheurebe or Pinot Gris, having F1-scores ranging between 62.6 and 92.5% (Cruz et al., 2019; Bendel et al., 2020a). However, previous studies either focused on a single cultivar or ignored cultivar effects, even though grapevine cultivars vary significantly in their morphological and physiological traits, such as intracellular compounds and surface structures like leaf hairiness and wax coating (Konlechner and Sauer, 2016; Gutiérrez-Gamboa et al., 2021; Herzog et al., 2021; Malagol et al., 2025), which can significantly affect spectral reflectance in the VIS-NIR range (Lacar et al., 2001; Diago et al., 2013; Gutiérrez et al., 2018; Mirzaei et al., 2019). Thus, in the present study a novel approach was applied to develop a model across multiple cultivars, in which a classification model of healthy samples was first developed to identify cultivar specific wavelengths before building the disease detection model. However, likely due to compensation by nearby or spectrally related regions within the 400–1,000 nm range, where high similarity and redundancy exist (Patro et al., 2024), the model was able to use alternative relevant wavelengths and still achieved a high F1-score of 98% for cultivar prediction. In contrast to the aim of reducing cultivar variability and improving disease classification specificity, the accuracy of the phytoplasma model was reduced. Although this approach did not successfully minimize the cultivar effect in the present study, it still presents a potential option for further research, particularly, in cases where the use of uniform plant material is not possible.

Due to the similarity of GY symptoms to those caused by various abiotic and biotic stresses on grapevine, distinguishing phytoplasma infections from other disease remains challenging. Cruz et al. (2019) showed that it is possible to distinguish among healthy, black rot, Esca, GY, and leaf blight infected leaves using various deep learning algorithms, achieving accuracies ranging from 98% to 99%. However, further differentiation among the three types of GY is necessary and even more critical for the application of trained models in quarantine monitoring. Al-Saddik et al. (2019) demonstrated that Flavescence dorée infection leads to an increase in anthocyanin content in black grapevine cultivars, whereas a decrease is observed in white cultivars. These changes in anthocyanin content are strongly correlated with reflectance at wavelengths: 690-710, 760-800, 1,050, 1,250, and 1,400 nm (Steele et al., 2009; Chen et al., 2015). Therefore, the disease classification models based on grapevine color developed in this study could account for pigment-related and associated chemical changes, which could reduce their confounding effects.

To make the field assessment process more efficient, a general model was developed that serves as a powerful tool to accelerate disease detection. The model in the present study achieved an F1-score of 97%, and importantly, class-specific important wavelength were identified. Interestingly, although FD is genetically closer to PGY than to BN (Krstić et al., 2022), the wavelength features identified in the present study suggest that the range from 806 to 820 nm was more important for distinguishing both BN and FD, whereas wavelengths between 680 and 688 nm contributed more significantly to the identification of PGY. For leaves infected with BN, two additional wavelengths at 400–403 and 784–787 nm were also identified as highly relevant. In contrast, Bendel et al. (2020a) reported that wavelengths around 459–492 and approximately 679 nm were important for identification of PGY, while 689 nm showed the highest relevance to BN. This study also highlighted differences in key wavelengths between greenhouse and field samples. Two key wavelength regions around 500 and 710 nm as being most important for the detection of Grapevine Leafroll-associated Virus were identified in a previous research (Bendel et al., 2020b). Consistent with the findings of Debnath et al. (2021), the present model successfully identified leaves affected by leafhopper, virus infection, and Fe- or Mg-deficiency. The key wavelengths associated with these stress factors were 806-811, 683-686/787, 680-686/820, and 814–820 nm for leafhopper, virus, Fe-deficiency, and Mg-deficiency, respectively. Most of these wavelengths are located in visible and near-infrared regions, which may strongly relate to the discoloration symptoms commonly observed in both biotic and abiotic diseases. While anthocyanins may not have played a determining role in the classification models due to the lack of consistently relevant wavelengths, other pigments such as chlorophyll a, chlorophyll b, and carotenoids, characterized by their absorption at 400–550 and 650–700 nm, as well as at red edge region, appear to have contributed significantly (Masoni et al., 1996; Peñuelas and Filella, 1998; Blackburn, 2007). The levels of these pigments may change differently in response to various infection or nutrient deficiencies. As reported from Masoni et al. (1996), chlorophyll a and b levels were significantly reduced in Mg- and Fe-deficient barley, wheat, corn, and sunflower plants, with the reduction being more pronounced in Fe-deficient plants than in those lacking Mg. Since the identified wavelength ranges fall within the visible and NIR regions, the disease identification model has potential for integration with multispectral sensor systems, which offer lower costs and faster processing speeds.

A proven and well-established chemometric approach based on mean spectra and PLS-DA is computationally efficient and can be trained robustly even when the number of samples per class is limited. Although the PLS-DA model is generally effective at distinguishing diseased leaves, false negatives for FD remain a challenge and require further optimization. Several studies have demonstrated that incorporating various vegetation indices, such as NDVI, NDRE etc. (Diago et al., 2013; Mahlein et al., 2013; Al-Saddik et al., 2017; Debnath et al., 2021) can improve classification accuracy. Therefore, integrating such indices into the present model may offer a promising direction for further improvement. In addition to spectral information, diagnostically relevant spatial features may provide complementary input to the model, potentially enhancing prediction specificity and overall accuracy (Sarić et al., 2022). Furthermore, different models, e.g. deep learning–based approaches, that utilize the full hyperspectral images, could be explored in future work.

## 5 Conclusions

The developed mobile platform enables flexible hyperspectral image acquisition both in the laboratory and in the field. The main output of the present study is the demonstrated first time the feasibility of the model in distinguishing Grapevine Yellow Diseases among biotic and abiotic stresses that have similar symptoms in both white and black grapevine cultivars, with a particular focus on FD infection. Models were developed and evaluated at three levels: i. for individual cultivars, ii. for groups of cultivars with similar berry skin color, and iii. for general application across cultivars. Leaves with symptoms of FD, BN, and PGY were able to differentiate both within and between cultivars. In addition, symptomatic leaves caused by Grapevine Leafroll-associated Virus, leafhopper infestation, Fe- and Mg-deficiency could also be distinguished from those affected by GYs. Key wavelengths associated with these stress symptoms were identified. These models hold strong potential as essential tools for vineyard monitoring, enabling early detection and eradication of FD infection to prevent disease dissemination.

## Data Availability

The raw data supporting the conclusions of this article will be made available by the authors, without undue reservation.
