# Supplementary material for: Hyperspectral differentiation of three grapevine yellows diseases and symptomatically similar stresses
Source: Front Plant Sci. 2026 Mar 19;17:1794713. doi: 10.3389/fpls.2026.1794713 (PMC13044154; doi:10.3389/fpls.2026.1794713)
Supplement: Supplementary file 1 [file DataSheet1.docx]

**Supplemental Materials**

**Table S1:** Number of images used for the phytoplasma models for single cultivars. Kerner (KER); Scheurebe (SRE); Chardonnay Blanc (CHA); Pinot Gris (PG); Non-symptomatic (C); Bois noir (BN); Palatinate grapevine yellows (PGY); Flavescence dorée (FD).

|  | **KER** | **SRE** | **CHA** | **PG** |
| --- | --- | --- | --- | --- |
| **C** | 40 | 60 | 40 | 40 |
| **BN** | 40 | 60 | 18 | 40 |
| **PGY** | 20 | 38 | - | - |
| **FD** | - | - | 40 | 33 |
| **sum** | **100** | **158** | **98** | **113** |

**Table S2:** Number of images used for the phytoplasma model for white grapevine cultivars (A) and its pro-processing model (B). Chardonnay Blanc (CHA); Kerner (KER); Pinot Gris (PG); Scheurebe (SRE); Non-symptomatic (C); Bois noir (BN); Palatinate grapevine yellows (PGY); Flavescence dorée (FD).

|  | **CHA** | **KER** | **PG** | **SRE** | **sum** |
| --- | --- | --- | --- | --- | --- |
| **C** | 20 | 20 | 20 | 20 | **80** |
| **BN** | 18 | 21 | 21 | 20 | **80** |
| **PGY** | 0 | 20 | 0 | 38 | **58** |
| **FD** | 47 | 0 | 33 | 0 | **80** |
| **sum** | **85** | **61** | **74** | **78** | **298** |

**Table S3:** Number of images used for disease model for white grapevine cultivars (A) and its pro-processing model (B). Aligote (ALI); Bacchus (BAC); Chardonnay Blanc (CHA); Kerner (KER); Pinot Blanc (PB); Pinot Gris (PG); Riesling (RIE); Scheurebe (SRE); Non-symptomatic (C); Bois noir (BN); Palatinate grapevine yellows (PGY); Flavescence dorée (FD); Fe-deficiency (Fe); Mg-deficiency (Mg); Leafhopper (LH); Grapevine leafroll-associated virus (V).

|  | **ALI** | **BAC** | **CHA** | **KER** | **PB** | **PG** | **RIE** | **SRE** | **sum** |
| --- | --- | --- | --- | --- | --- | --- | --- | --- | --- |
| **C** | 10 | 10 | 10 | 10 | 10 | 10 | 10 | 10 | **80** |
| **BN** | 0 | 0 | 16 | 16 | 0 | 16 | 16 | 16 | **80** |
| **PGY** | 0 | 0 | 0 | 20 | 0 | 0 | 0 | 38 | **58** |
| **FD** | 0 | 0 | 47 | 0 | 0 | 33 | 0 | 0 | **80** |
| **Fe** | 0 | 0 | 0 | 0 | 80 | 0 | 0 | 0 | **80** |
| **Mg** | 0 | 40 | 0 | 0 | 0 | 0 | 40 | 0 | **80** |
| **LH** | 0 | 0 | 0 | 0 | 0 | 0 | 80 | 0 | **80** |
| **V** | 40 | 0 | 40 | 0 | 0 | 0 | 0 | 0 | **80** |
| **sum** | **50** | **50** | **113** | **46** | **90** | **59** | **146** | **64** | **618** |

**Table S4:** Number of images used for disease model for black grapevine cultivars (A) and its pro-processing model (B). Cabernet Dorsa (CD); Donrfelder (DOF); Dunkelfelder (DUF); Pinot Meunier (PM); Pinot Noir (PN); Non-symptomatic (C); Bois noir (BN); Fe-deficiency (Fe); Mg-deficiency (Mg); Leafhopper (LH); Grapevine leafroll-associated virus (V).

|  | **CD** | **DOF** | **DUF** | **PM** | **PN** | **sum** |
| --- | --- | --- | --- | --- | --- | --- |
| **C** | 20 | 20 | 20 | 20 | 20 | **100** |
| **BN** | 34 | 33 | 0 | 33 | 0 | **100** |
| **Fe** | 0 | 0 | 0 | 0 | 99 | **99** |
| **Mg** | 0 | 0 | 99 | 0 | 0 | **99** |
| **LH** | 0 | 0 | 100 | 0 | 0 | **100** |
| **V** | 0 | 0 | 0 | 0 | 99 | **99** |
| **sum** | **54** | **53** | **219** | **53** | **218** | **597** |

**Table S5:** Number of images used for disease model for white and black grapevine cultivars (A) and its pro-processing model (B). Aligote (ALI); Bacchus (BAC); Cabernet Dorsa (CD); Chardonnay Blanc (CHA); Donrfelder (DOF); Dunkelfelder (DUF); Kerner (KER); Pinot Blanc (PB); Pinot Gris (PG); Pinot Meunier (PM); Pinot Noir (PN); Riesling (RIE); Scheurebe (SRE); Non-symptomatic (C); Bois noir (BN); Palatinate grapevine yellows (PGY); Flavescence dorée (FD); Fe-deficiency (Fe); Mg-deficiency (Mg); Leafhopper (LH); Grapevine leafroll-associated virus (V).

|  | **ALI** | **BAC** | **CD** | **CHA** | **DOF** | **DUF** | **KER** | **PB** | **PG** | **PM** | **PN** | **RIE** | **SRE** | **sum** |
| --- | --- | --- | --- | --- | --- | --- | --- | --- | --- | --- | --- | --- | --- | --- |
| **C** | 7 | 7 | 6 | 6 | 6 | 6 | 6 | 6 | 6 | 6 | 6 | 6 | 6 | **80** |
| **BN** | 0 | 0 | 10 | 10 | 10 | 0 | 10 | 0 | 10 | 10 | 0 | 10 | 10 | **80** |
| **PGY** | 0 | 0 | 0 | 0 | 0 | 0 | 20 | 0 | 0 | 0 | 0 | 0 | 38 | **58** |
| **FD** | 0 | 0 | 0 | 47 | 0 | 0 | 0 | 0 | 33 | 0 | 0 | 0 | 0 | **80** |
| **Fe** | 0 | 0 | 0 | 0 | 0 | 0 | 0 | 40 | 0 | 0 | 40 | 0 | 0 | **80** |
| **Mg** | 0 | 27 | 0 | 0 | 0 | 27 | 0 | 0 | 0 | 0 | 0 | 26 | 0 | **80** |
| **LH** | 0 | 0 | 0 | 0 | 0 | 40 | 0 | 0 | 0 | 0 | 0 | 40 | 0 | **80** |
| **V** | 27 | 0 | 0 | 27 | 0 | 0 | 0 | 0 | 0 | 0 | 26 | 0 | 0 | **80** |
| **sum** | **34** | **34** | **16** | **90** | **16** | **73** | **36** | **46** | **49** | **16** | **72** | **82** | **54** | **618** |


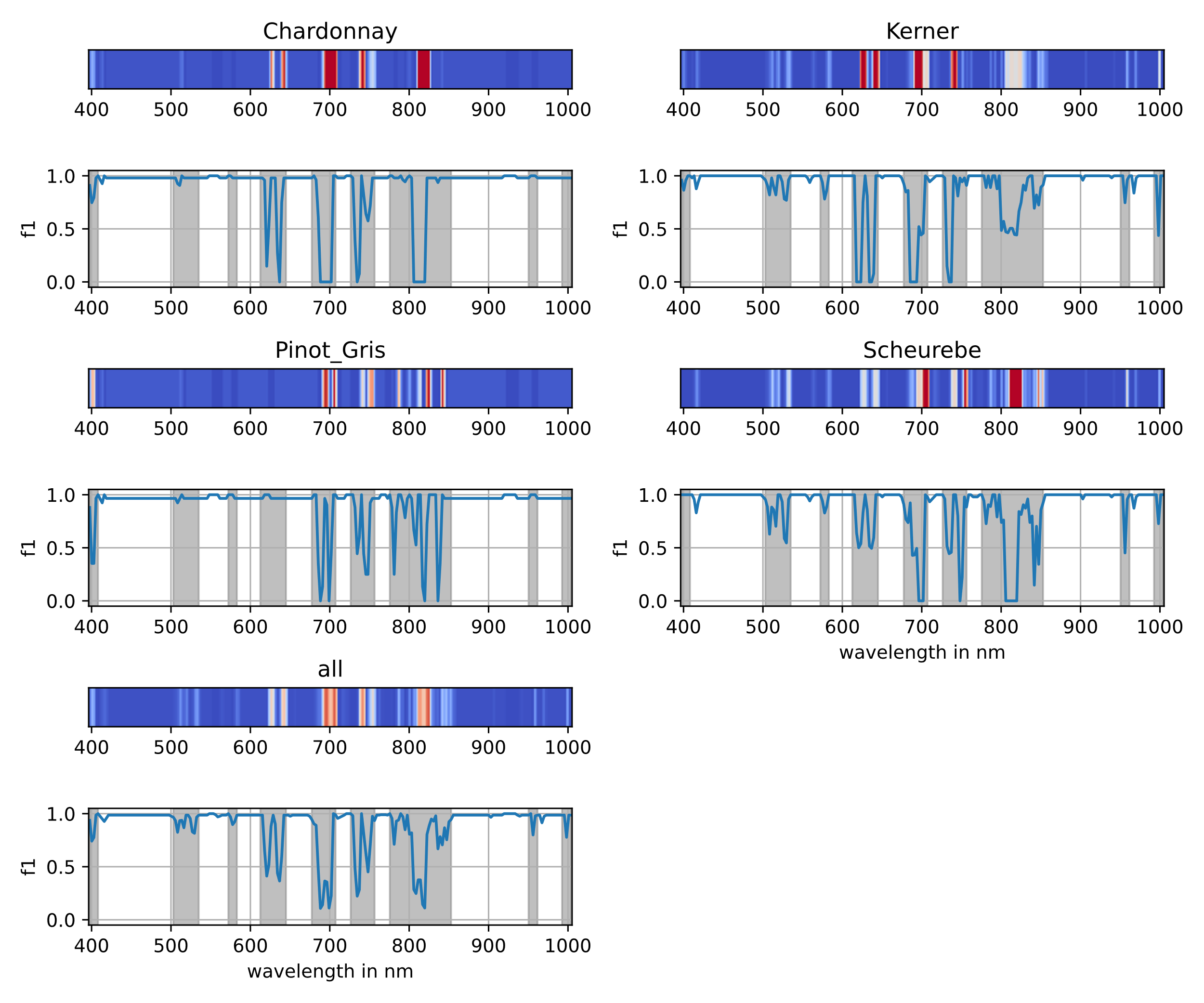


**Figure S1:** Contribution map of the individual wavelengths for the cultivar classification performance of the model that was trained on control leaves of these four cultivars. The blue line shows the F1-score on the test set if the individual wavelength value was replaced. The gray area marks the wavelengths where the overall F1-score was reduced by more than 10% with respect to the unmodified data.
